# Welfare Challenges of Dairy Cows in India Identified Through On-Farm Observations

**DOI:** 10.3390/ani10040586

**Published:** 2020-03-31

**Authors:** Siobhan Mullan, Surej J. Bunglavan, Elizabeth Rowe, David C. Barrett, Michael R. F. Lee, Deepa Ananth, John Tarlton

**Affiliations:** 1 School of Veterinary Sciences, University of Bristol Langford, Somerset BS40 5DU, UK; lizzie.rowe@bristol.ac.uk (E.R.); David.Barrett@bristol.ac.uk (D.C.B.); michaelrf.lee@bristol.ac.uk (M.R.F.L.); john.tarlton@bristol.ac.uk (J.T.); 2 College of Veterinary and Animal Sciences, Department of Animal Nutrition, Kerala Veterinary and Animal Sciences University, Pookode, Wayanad District, Kerala 673576, India; surej.joseph@kvasu.ac.in (S.J.B.); adeepa@kvasu.ac.in (D.A.); 3 Rothamsted Research, North Wyke, Devon EX20 2SB, UK

**Keywords:** animal welfare, dairy cow, India

## Abstract

**Simple Summary:**

India has the largest population of dairy cattle in the world, but little work has been done to objectively assess their welfare. Formal welfare assessment is needed to identify any welfare problems and inform solutions to these problems. Dairy cattle on 38 farms in Kerala, India, were observed using standardised welfare assessment protocols. The major welfare problems identified in this study were that all cows were tied to their housing on a rope < 1 m that attached to a halter that pierced the nasal septum, most farms did not provide cattle with unlimited access to water, and access to food was also limited. On half the farms, cattle were tied up inside for the whole day, and most of those given outdoor access were also tied when outside. These results show the need to encourage dairy farmers in India to stop tying their cattle, and to provide them with unlimited access to drinking water and a readier supply of a good healthy diet. Welfare assessment protocols were used successfully in this study, suggesting that they can and should be used regularly to assess cattle welfare in India. Making these changes could substantially improve the welfare of tens of millions of cattle.

**Abstract:**

India has the largest population of dairy cattle in the world at over 48 million animals, yet there has been little formal assessment of their welfare reported. Through observations of dairy cows on 38 farms in Kerala, India, we aimed to investigate the welfare of these animals and the practicality of animal-based assessments within common farming systems. Substantial welfare challenges were identified. All cows were close-tied (less than 1 m length) via a halter that pierced the nasal septum when housed, which was for the entire day (50% of farms) or part thereof. When outside access was available, it was also usually restricted by close-tying, longline tether, or hobbling. Ad libitum water was only available on 22% of farms and food access was also restricted (mean of 4.3 h/day). Future work should focus on encouraging dairy farmers in India to improve the welfare of their dairy cattle by: ceasing to tie and tether cattle (or at least providing tied and tethered cattle with exercise opportunities); providing unlimited access to drinking water and a readier supply of food (especially quality green forage/fodder); cleaning housing more frequently; providing strategies to prevent heat stress; breeding cattle suited to environmental conditions and with increased resistance to heat stress; and carrying out welfare assessments more regularly using a validated protocol and rectifying the causes of poor welfare. Such changes could substantially improve the welfare of tens of millions of cattle.

## 1. Introduction

India is the world’s largest milk-producing country, with a 20% share in global production [1]. Milk production in India differs from most countries in that more than half of the milk produced is from buffaloes (*Bubalus bubalis*), followed by indigenous cows (*Bos indicus*) and crossbred cows [2]. Since the 1960s, indigenous cows have been cross-bred with European breeds (*Bos taurus*) with higher productivity, such as the Holstein, Friesian, Jersey, Brown Swiss and Red Dane, to increase milk production [3]. Despite less than half of India’s milk being produced from cows, the country has the largest dairy cow population in the world: in 2016, this was 48,610,350 dairy cows—17.8% of the world’s total population [4]. Indian dairy farming mainly consists of traditional smallholder production systems, although the number of larger commercial systems is growing [5].

Much of the Indian population follow Hinduism, a religion that holds cattle sacred; consequently, most states do not permit the slaughter of dairy cattle for meat and non-productive animals may be housed in shelters known as gaushalas until they die of natural causes [6]. Some states permit only the slaughter of bullocks and/or water buffalo, and a few permit the slaughter of all bovines, including Kerala state. Despite their sanctity, and despite having the largest population of dairy cows in the world, there are no regulations safeguarding dairy cow welfare in the country. In 2014, the international animal welfare organisation World Animal Protection and India’s National Dairy Research Institute released a National Code of Practices for the Management of Dairy Animals in India (the National Dairy Code) [7]; these organisations have been calling on the Indian government to implement and enforce this code, but engagement and uptake has been slow. Thus, assessment of dairy cow welfare is not routinely (if ever) carried out. 

There appears to be a lack of published peer-reviewed studies on the welfare of dairy cows in India, and on the assessment of dairy cow welfare using scientifically validated assessment protocols. Sharma et al. [6] recently assessed the welfare of cows in gaushalas using both animal-based and resource-based measures. Shelters were found to consist of sheds for housing cows, predominately with loose or free-stall housing but some with tethered stalls, and most gave cows access to a yard. The authors identified several welfare problems related to the inadequacy of the facilities, including insufficient space per cow, poor floor quality, restricted movement and lack of grazing. Publications pertaining to on-farm assessment of cows remain lacking. In a recent review, Kumar et al. [5] concluded that “so far little effort has been made in India to understand dairy animal welfare or to identify the indicators of welfare or to assess the level of welfare. Therefore, the status of dairy animal welfare under our different dairy farming systems needs to be studied so that the animal welfare areas or management practices which jeopardize animal welfare could be identified and a strategy could be developed for enhancing the animal welfare.” Regular assessment of dairy cow welfare in India, through identifying and rectifying causes of poor welfare, could improve the lives of tens of millions of animals. Improving welfare can also benefit farmers by improving productivity and profitability, for example, through reduced animal mortality and improved health, improved product quality, improved disease resistance, reduced medication use, lower risk of zoonoses and foodborne diseases, and the potential to command higher prices from consumers [8].

Protocols to evaluate and monitor the welfare condition of farm animals have been developed and scientifically validated. For example, Welfare Quality® was the largest ever European research project on animal welfare, which produced standardised measures of welfare criterion integrated into assessment protocols [9]. The Welfare Quality® assessment protocols have been developed to be practical to implement and able to be performed quickly and accurately by anyone following a short training period [10].

The aim of this study was to investigate the current welfare state of dairy cows in India, based on a sample in the state of Kerala, where cattle slaughter is permitted, and further, to assess whether these welfare measures are practical for use in Indian dairy farming systems.

## 2. Materials and Methods 

### 2.1. Farm Visits

Thirty-eight farms were visited during December 2016 in five geographical regions of Kerala within 90 km of Thrissur. The regions were chosen to represent a broad range of climatic and farming practices: Vannamada (dry lands), 15 farms; Thrissur (wetlands), 12 farms; Guruvayur (coastal area), 5 farms; Kunnamkulam (mid lands), 4 farms; Peechi (hill range), 2 farms. Farms were already involved in a range of outreach interventions by the Kerala Veterinary and Animal Sciences University (KVASU). Two members of the research team visited each farm at a time between 07:00 and 18:00: S.M. conducted animal and resource-based observations and asked the farmer about elements not obtainable by direct observations, with translation provided by a native speaker (S.J.B. or another). S.J.B. also collected additional data not presented here.

### 2.2. Animal-Based Observations

Where possible, every cow, milking or dry, on each farm was observed for physical and behavioural measures by a single observer throughout (S.M.), using standardised protocols from Welfare Quality [11] and AssureWel [12] where suitable. Young stock and bulls were not observed. Almost all cows were observed whilst close-tied in the housing. On a small number of farms some cows were inaccessible, for example, in a distant field. At times, individual measures could not be observed on an individual cow, for example, lying cows were not made to rise, therefore lameness could not be assessed. All other measures were easily observed for lying cows, apart from Body Condition Score (BCS) and rumen fill; therefore, these measures were only recorded for lying cows when the observer was confident in making a true assessment. On larger farms, where there was insufficient time to observe all cows, the total number of cows observed was determined by the time available, and a randomised sampling strategy of observing every nth cow was employed to eliminate sampling bias; for example, if on a farm of 150 cows, time availability dictated taking a sample of 15 cows, every 10th cow was observed. The protocol was devised by S.M. and S.J.B., and observations made were as follows.

- Lameness, being absent or present (in tied animals if showing ‘resting a foot, standing on the edge of a step, frequent weight shifting between feet, reluctance to bear weight on a foot when moving’, or in non-tied animals if showing ‘imperfect temporal rhythm in stride creating a limp’) [11]. Approximately 3 min was allocated to evaluate each cow in order to allow sufficient time for any signs of lameness to become apparent.
- Body condition—being thin, moderate or fat [11]—determined by observation.
- Dirtiness was assessed on one side of the animal only: score 0 (<15 cm manure), score 1 (15–40 cm manure), score 2 (>40 cm manure).
- Hair loss, lesions or swellings—all assessed on each of the four body regions (1 side only): head/neck, body, forelimb, and hindlimb [12] (score 0 (<2 cm), score 1 (2–5 cm), and score 2 (> 5 cm)).
- Broken tail (absent/present)—presence was determined by a clear deviation from a straight line in the tail.
- Diarrhoea (absent/present).
- Ocular discharge (absent/present).
- Nasal discharge (absent/present).
- Rumen fill (score 0 (no depression), score 1 (slight depression), and score 2 (deep depression)).
- Panting (score 0 (respiration rate < 41/min), score 1 (respiration rate 41–70/min), and score 2 (respiration rate > 70/min)).
- General demeanour (calm, uneasy, and frightened).
- Posture during observation (standing/lying).

In addition, it was noted whether tongue rolling was seen being performed by any animal on the farm during the visit. 

### 2.3. Resource-Based Measures

**Figure 1:**
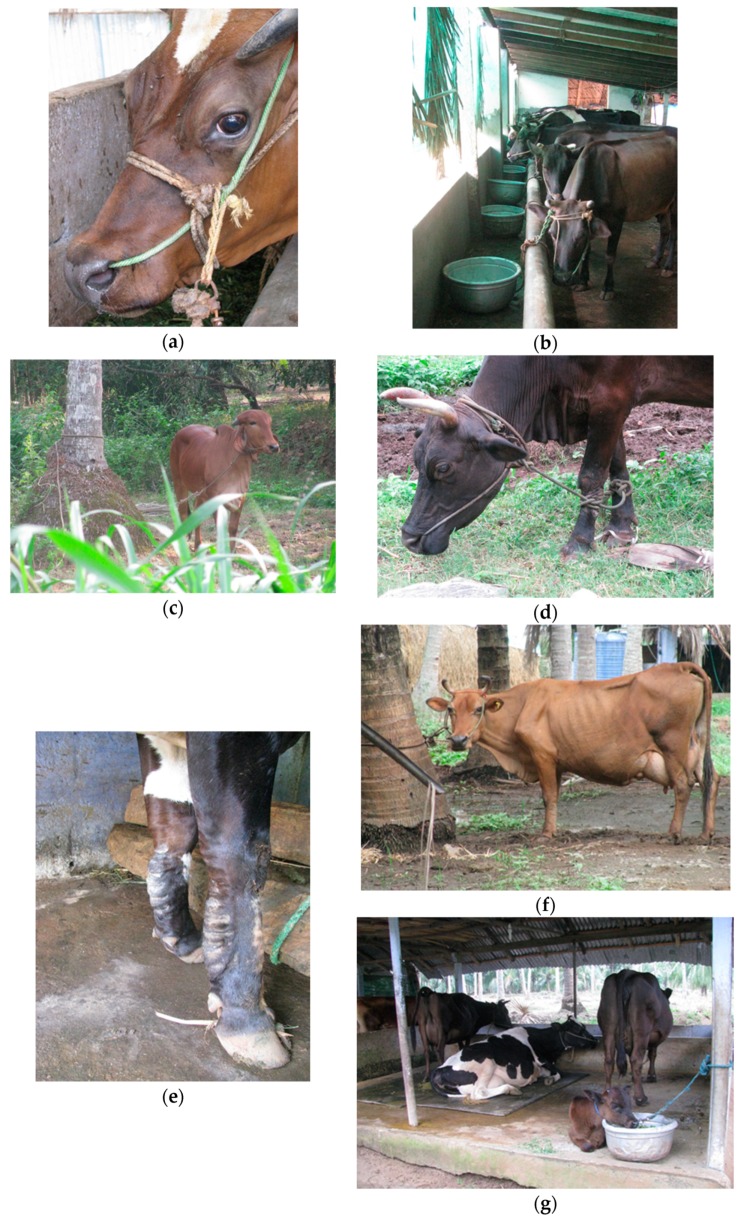
(**a**) The halter used on all cows, (**b**) close-tied in the shed, (**c**) longline tether, approx. 10 m (*Bos indicus* heifer not in study population, choosing to stand near tree), (**d**) hobbled cow, (**e**) hobbling injuries, (**f**) close-tied outside, and (**g**) rubber mat under one cow.

Farmers were asked to describe the daily routine for their cows—information was recorded about the number of hours/day that cows spent close-tied (<1 m); hobbled from the head to both forelimbs (see Figure 1); tethered (>1 m) (tether length noted); free (neither tethered nor tied); walked in hand; in the shed or outside. In addition, information was collected about water provision (number of hours that water was provided ad libitum/number of ‘drinks’ offered when not ad libitum), number of hours that any food (concentrate or roughage) was available to the cow, frequency of milking, average milk yield of the herd and flooring type. Information on individual milk yield and veterinary treatments was not available.

Whilst on the farm, the presence of water, feed and fodder (including type), rubber lying mat, fans, misting system and halter type were noted for each cow.

### 2.4. Data Analysis

Data were analysed using SPSS vs. 21 at both the individual cow level and the farm level. As many of the farms had fewer than 10 cows, it was considered that analysis of the relationship between measures should be conducted at the cow level. Pearson’s Chi-square test was utilised for relationships on an individual cow between the presence and absence of lameness, dirt (any amount), hair loss, lesion, swelling (all any size), panting (any rate), deep ruminal depression, thin, fat, and uneasy/frightened cattle. These measures were also compared for cows that had access to the outside versus those that did not, and measures of cow comfort (lameness, dirtiness, hair loss, lesions and swellings) were compared for cows that were and were not housed on rubber mats using Pearson’s Chi-square test. Finally, the relationships between outcome measures and number of cows on the farm, average milk yield per farm, number of hours spent in the shed, were tested using the Mann–Witney test. At the farm level, the relationship between farm attributes was explored by using Spearman’s rho correlations for farm size, number of hours cows spent in the shed, number of hours spent close-tied, number of hours water was available, number of hours food was available, and average milk yield.

## 3. Results

The 38 farms contained 571 cows in total and ranged in size from 2 to 150 cows (mean 15, median 8). The observed cattle on all farms were visually *B. taurus* and mostly a mixture of breeds derived from historically imported stocks of Holstein, Jersey and Brown Swiss cattle, with some crossbreeding with indigenous breeds of *B. indicus*, which was evident on occasions through drooping ears or a neck hump. Kerala’s cattle population consists of 82% crossbreeds [13]. The native Vechur cattle in Kerala are the smallest breed in the world and therefore not suitable for high milk production.

**Figure 2:**
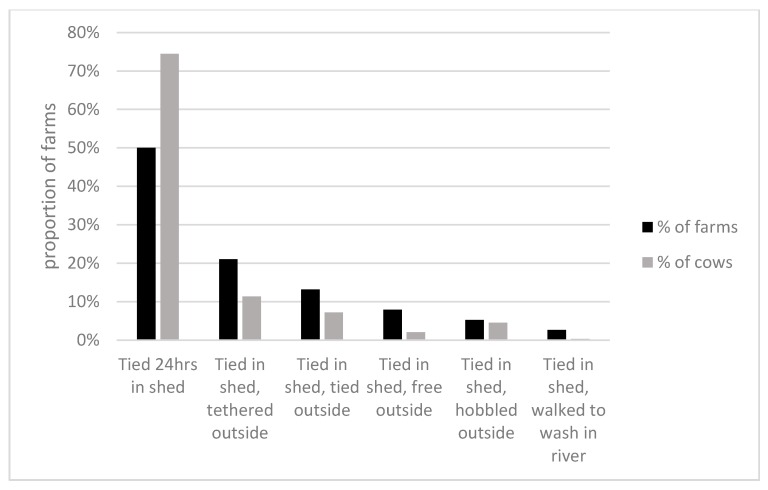
The different farm systems in use in the study.

All cows wore a halter that penetrated the nasal septum and went behind the ears (see Figure 1). All cows spent a minimum of 12 h close-tied in the shed. On 19 farms (50%), owning 75% of the cows in the study, the cows spent 24 h a day close-tied in housing. For those farms where the cows had outside access for part of the day, eight farms (21% of farms or 11% of cows) used long-line tethers of between 3 and 20 m (mean 10 m) for between 2 and 12 h (mean 5.6 h); on five farms (13% of farms or 7% of cows), the cows spent 2 h close-tied outside; on three farms with 2% of the cows, the cows were free in an outside grazing space for 2, 6 and 10 h, respectively; on two farms with 5% of cows, the cows were hobbled for 6 h, and on one farm, the two cows were walked out in hand daily for 30 min to the river to wash (See Figure 2). 

Of the 32 farms for which information was available, 16 farms (50%) received water only in discrete drinks (range 2 to 6 times, mean 3.4 times a day), seven (22%) farms had ad libitum access to water, and nine (28%) farms provided ad libitum water for part of the day; of these nine farms, six farms also provided two additional drinks and one farm provided one additional drink per day. During the observations, water was present on 12 out of 35 farms (34%) and food was present on 11 out of 36 farms (31%). On three farms, this food was rice (*Oryza sativa*) straw; on another three farms, this was ‘porridge’, a concentrated meal of either wetted commercial pellets or a mixture of food stuffs such as spent wet distillers’ grains, tapioca starch waste, soy hull, green pea husk, (non-conventional feed resources available in Kerala), banana (*Musa spp.*) skins and rice husks; on two farms, this food was chopped fodder grass; on another two farms, the cows were grazing. Farmers (n = 18) estimated that food (concentrate or forage) was available to cows for a mean of 4.3 h per day (range 2 to 11 h). 

The flooring on almost all farms (35/38, 92%) was concrete and one farm used tiles, another soil, and another a mixture of soil and concrete. There were rubber mats available to at least some cows on 18/34 farms (53%), fans present on six farms (16%) and a misting cooling system on one farm. All farmers milked their cows twice a day and the mean of farmer-reported milk yields at the time was 9.4 L/cow/day, range 5–20 L/cow/day (n = 14 farms).

**Figure 3:**
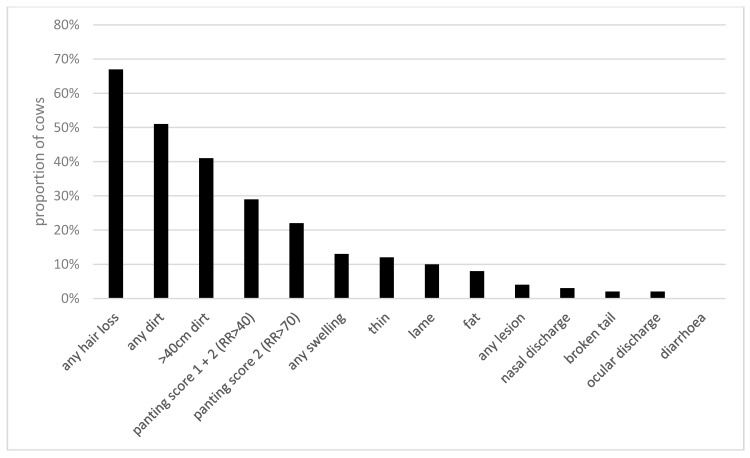
The proportion of cows recorded with each measure (n = 169–391).

**Figure 4:**
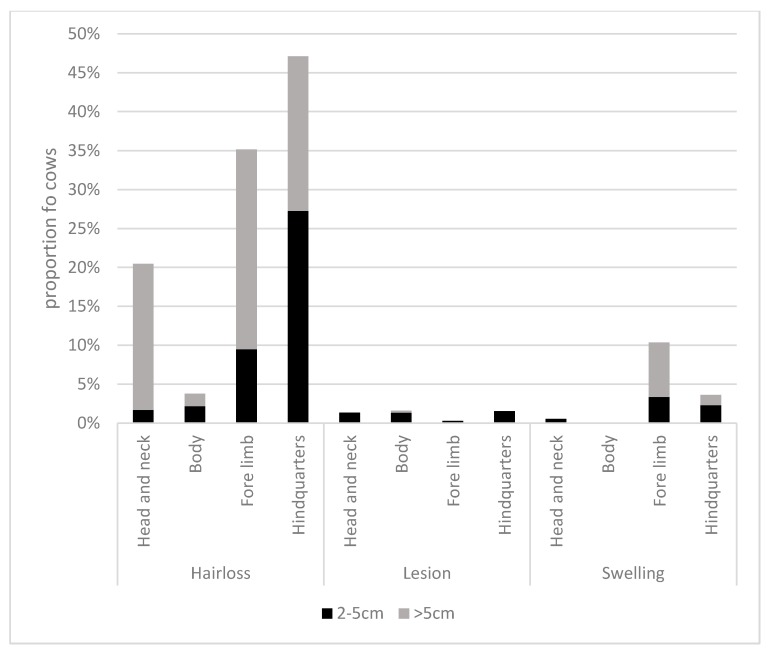
The proportion of cows recorded with hair loss, lesions and swellings in different body regions.

A total of 405 individual cows were observed, ranging from 2 to 59 per farm. On 28 farms (74%), all cows were observed; on other farms, the following numbers were observed: 2/5, 2/7, 25/150, 11/16, 4/8, 13/16, 10/12, 59/70, 6/10, and 6/10. Where n is not specified in the following reports, at least 325 cows were observed for that measure. Overall, 85.5% of cows were standing rather than lying during their observation, and 68.1% of 257 cows were on a rubber mat. Most cows were classified as calm (86.2%), with 10.2% and 3.6% recorded as uneasy and frightened, respectively. Welfare outcomes are shown in Figure 3. Lameness was observed in 35/335 cows (10.4%) and 51.3% of cows were dirty—of which, 41.2% were score 2. A minority of cows were recorded as thin (12.2%) or fat (8.0%). In total, 68.9% of cows had either hair loss, a lesion or swelling—of which, 67.1% had hair loss, 4.3% had a lesion and 12.9% had a swelling somewhere on their body. Further detail is provided for hair loss, lesions and swellings in Figure 4. The most common region for hair loss was the hindquarters (47.0%), although the most common large areas of hair loss (>5 cm) were seen on the forelimb (25.7%). Swellings were most frequently observed on the forelimb (3.4% were between 2 and 5 cm and 7.0% > 5 cm). Lesions were infrequently recorded (<2% for all regions). There were 2.4% of cows recorded with a broken tail, 2.9% with nasal discharge, 1.6% with ocular discharge, 6.8% with a deep rumen depression, 7.1% with no rumen depression and no cows with observable signs of diarrhoea (n = 169). Almost 29% of cows were showing signs of heat stress, as 7.0% and 21.7% of cows were recorded with a panting score of 1 (mild heat stress) and 2 (moderate heat stress), respectively. Stereotypical tongue rolling was observed in at least one animal on each of the three farms (8%).

Investigations of the relationships between measures showed that lame cows were more likely to be observed panting (*p* < 0.001) but less likely to be recorded as dirty (*p* = 0.016). In addition, cows recorded as dirty were more likely to have at least one area of hair loss (*p* = 0.001). Hair loss and swellings were positively associated (*p* < 0.001) but swellings were less likely to be observed in panting (*p* = 0.026) or thin (*p* = 0.016) cows. Thin cows were less likely to have hair loss (*p* = 0.037) but more likely to be observed panting (*p* = 0.021). Cows that spent any time outside were less likely to be observed as lame (*p* = 0.001), dirty (*p* = 0.034), with hair loss (*p* = 0.026), with a deep ruminal depression (*p* < 0.001), panting (*p* = 0.001) and thin (*p* = 0.012), but more likely to be recorded with a swelling (*p* < 0.001) and as uneasy/frightened (*p* = 0.003). Cows housed on a rubber mat were more likely to be recorded as lame (*p* < 0.001), panting (*p* < 0.001) and thin (*p* = 0.005) but less likely to be dirty or have a swelling (both *p* < 0.001). Larger farms were associated with more dirt and hair loss being recorded (both *p* < 0.001) but fewer uneasy/frightened cows (*p* = 0.005). The longer cows spent in the shed, the more likely they were to be recorded as lame (*p* = 0.006), thin (*p* = 0.016) and with hair loss (*p* = 0.004), but less likely to have swellings (*p* = 0.001) and be uneasy/frightened (*p* = 0.003). Thin cows were more likely to be recorded on farms with a higher mean milk yield (*p* = 0.028).

The number of adult cows on the farm was significantly positively correlated with number of hours cows spent in the shed (*r* = 0.373, *p* = 0.030), number of hours cows spent close-tied (*r* = 0.398, *p* = 0.016) and milk yield (*r* = 0.804, *p* = 0.001). As all cows were close-tied when in the shed and some were additionally close-tied outside at times, the number of hours cows spent in the shed was also significantly positively correlated with the number of hours spent close-tied (*r* = 0.945, *p* < 0.001). Water was rarely available out of the shed and food was often eaten quickly when provided indoors and may, or may not, have been available outside. The number of hours spent in the shed and the number of hours close-tied were positively correlated with the number of hours water was available (*r* = 0.709, *p* = 0.003 for both) and average milk yield (*r* = 0.64, *p* = 0.014 for both), but negatively with the number of hours food was available (*r* = −0.897, *p* < 0.001, *r* = −0.900, *p* < 0.001, respectively). There was an inverse relationship between the number of hours food and water were available (*r* = −0.618, *p* = 0.024) and the number of hours food was available was negatively correlated with average milk yield (*r* = −0.771, *p* = 0.015).

## 4. Discussion

To the authors’ knowledge, this study was the first to investigate the on-farm welfare state of dairy cows in India using a robust assessment protocol, and to test the plausibility of using such measures, mostly from the Welfare Quality® protocol [11], on these types of farm. Despite being small in scale and restricted to one state (Kerala), the study has revealed clear welfare issues and highlights areas where future work is needed to improve dairy cattle welfare in India. 

Although the study looked at a range of different farm sizes (2–150 cows), the farm sizes were small (mean 15 cows, median 8) on average. Anecdotally, it was suggested that there was a trend for larger, wholly indoor farms becoming more common, and we found that larger farms were associated with more time spent indoors and being close-tied. All cows spent at least half of the day close-tied inside housing, and three-quarters of the cows spent all day close-tied inside. In this study, all cows were fitted with a rope halter that pierced the nasal septum, which results in pressure on sensitive tissues throughout the life of the cow and, if carried out without analgesia, would be painful. Tie-stall housing systems for dairy cows are used in many parts of the world; however, the prevalence in this study (100%) was higher than the prevalence reported in other countries. In Europe, between 20% (in lowland areas) and 80% (in upland areas) of cows were reportedly tethered at least during the winter [14]. Tie stalls were the primary housing type for lactating cows on 39% of farms in the USA in 2014 [15]. Several countries have introduced bans on building new tie-stall systems, and complete bans are also being introduced—for example in Sweden, where approximately 32% of dairy cows were in tie-stall systems in 2015, the construction of new tie-stall barns has been banned since 2007 [16]. Likewise, in Norway, building new tie-stall barns is banned and a complete ban on tie-stalls is scheduled for 2034 [17].

The welfare of cows that are tied is considered compromised because the cow’s movement is restricted, leading to difficulties in changing position, grooming, adopting normal resting postures, carrying out normal social interactions, and exercising [18]. Therefore, tied cows are prevented from the freedom to express normal behaviour—one of the ‘five freedoms’ of animal welfare [19]—suggesting that a basic need of the cattle observed in this study was denied. Recently, Lundmark Hedman et al. [16] found that the incidence of non-compliance with either legislation or private standards on animal welfare on farms in Sweden was higher in farms with tie-stall housing than in free-stall (cubicle) systems, indicating their limited potential for good cow welfare; the authors concluded that a total ban on tie-stalls for dairy cows would have a positive impact on cow welfare. Popescu et al. [20] identified several welfare problems in tied-housing systems, including poor body hygiene and mastitis—the latter being “at an alarming level”—as well as poor scores for positive emotional state (assessments were conducted using the Welfare Quality® protocol). In the same study, the welfare problems identified were more severe in tie-stall farms without access to free exercise than those that provided turnout into a paddock, pasture or both, suggesting an ameliorating effect on animal welfare of providing exercise opportunities. However, in the present study, of the cows that were given some outdoor access (25%), only 2% had free access to an outdoor grazing space; the rest were either tethered, hobble-tied or close-tied outdoors. Therefore, even cows with outdoor access had limited opportunity for exercise and natural behaviours associated with free movement, and three-quarters of the cows in the study had no exercise. We found outside access to be protective against lameness, dirtiness, hair loss, panting and being thin. However, outside access was associated with much higher levels of swellings, due to the large swellings that were observed on the forelimbs of all cows that were hobbled (see Figure 1). Hobbling did provide walking and grazing access but in addition to the physical injuries, behaviour restriction was also significant as the head could not rise above the height of the shoulder.

Most cows on the farms in this study could have been kept untethered as they were kept in contained environments or requiring minimal effort and resources to make them secure. Conversations with farmers suggested that as well as the risk of escape and concern about injuries to free-moving cattle, a major barrier appeared to be the confidence of stock-keepers around untethered cattle. Therefore, training would be required to help stock-keepers gain confidence in handling cattle in order to encourage farmers to stop tying and tethering their animals and therefore improve their welfare.

Only 22% of the farms for which information was available gave cows ad libitum access to water, and only 34% of farms had water present during observations. Freedom from thirst by ready access to fresh water is one of the ‘five freedoms’ of animal welfare: guidelines which focus on the avoidance of unnecessary suffering [19]. Therefore, the farms in this study were not meeting one of the basic requirements to ensure avoidance of suffering. Unlimited access to drinking water is especially important in hot climates such as India, and particularly for dairy cattle, which have a high water intake requirement [21]. In addition, only 31% of farms had feed/fodder present during observations, and food was available to cows for a mean of 4.3 h per day—sometimes for as little as 2 h a day. As freedom from hunger by ready access to a diet to maintain full health and vigour forms the first of the ‘five freedoms’ alongside freedom from thirst [19], this result again suggests that the basic needs of the cattle were not always being met. In addition, it is likely that milk production and rumen health would be impacted due to the lack of ad libitum roughage and water and suitable diet and 6.9% of cows demonstrated a substantial lack of rumen fill. The lack of association between availability of water and milk yield may have been a result of the relatively small sample size and lack of variation in each measure or other confounding factors such as management factors and breed which also influence milk yield. Finally, the lack of roughage may have contributed, along with the behavioural restrictions, to the stereotypical tongue rolling seen on a small proportion of farms [22]. 

The finding that most cows were not given ad libitum access to drinking water is especially worrying given that almost 29% of cows were showing signs of heat stress, observed as an increase in respiration rate to aid cooling through respiratory evaporation: 7.0% of cows had a respiration rate of 40–70 breaths/minute, considered as panting in cattle and reflecting mild heat stress [23], and 21.7% of cows had a panting score of 2 (over 70 breaths/minute), reflecting at least moderate heat stress [23]. Access to drinking water is essential to improve conditions for heat-stressed cattle [24]. Most farms had no other systems in place to help prevent or ameliorate heat stress; only 16% of farms had fans and only one farm had a misting cooling system.

The average farmer-reported milk yield (9.4 L/cow/day) was slightly lower but comparable to European breeds such as the Jersey or Guernsey (approximately 13 L/cow/day) [25]. Average farmer-reported yields also ranged up to 20 L/cow/day, which is similar to Friesians (approximately 23 L/cow/day), although lower than Holsteins (up to approximately 33 L/cow/day) [25]. High-yielding breeds are more prone to suffering from heat stress [24]. However, in our study, we found that higher milk yield was only associated with being thin. The number of hours food was available was negatively correlated with average milk yield. The cows that grazed outdoors, and which therefore had higher hours of food availability, were from smaller farms with genetically inferior cattle, which may have led to this negative correlation. 

The fact that native breeds have been crossed with high-yielding breeds may have led to cross-breeds in India that are maladapted to the diet available, leading to poor body condition [26]. More work is needed to confirm this finding, but it suggests that future breeding programmes should focus on balancing selection for improved productivity with adaptation to environmental conditions and resistance to heat stress [26,27].

Lameness was observed in 10.4% of cows. This is similar to the prevalence of lameness observed in tie-stall farms in Switzerland both with access to exercise opportunities (9.6%) and no exercise opportunities (13.2%) [28]. The average incidence of lameness in dairy cattle is approximately 35% of cows annually, ranging from 5% to as high as 60% [2]. That the lameness prevalence in this study was at the lower end of this spectrum is a positive finding; however, the incidence as measured over a year may be higher, and the sample size was relatively small, meaning that this may not be a reliable estimate of lameness prevalence in Indian dairy cattle. In addition, lying cows, which are more likely to be lame [29], were not made to rise, again potentially leading to an underestimation of prevalence. That lameness was more likely to be observed in cows housed on rubber mats was contrary to expectations and local veterinary advice. However, it may have been that farmers were proactively providing mats for cows that were lame, or that other factors pre-disposing to lameness were stronger than any mitigating effect of the mat. A risk factor for lameness identified in this study was increased hours spent in the shed or close-tied (which were themselves highly correlated) and it may be that the lack of exercise contributed to lameness development. The feeding of high-energy protein feed supplements to maximise the milk production along with a concomitant low supplementation of quality green fodder is also likely to have contributed to lameness of dairy cattle in Kerala [30]. 

The results suggest that the cows were generally in good health and had a calm demeanour; most cows were classified as calm, the prevalence of health issues such as a broken tail, nasal and ocular discharge and rumen depression ranged between 1.6% and 7.1%, and no cows had observable signs of diarrhoea. These encouraging findings may be because cow slaughter is permitted in the state of Kerala, enabling sick cows to be culled (although the number of cows culled on the observed farms in this study is not known). 

A further encouraging finding is that the majority (79.8%) of cows had a normal body condition score (BCS). This exceeds the percentage of dairy cows with good body condition in a similar geographic region (Bangladesh), where 65.5% of cows were found to have an ideal body condition score [31]. It is also better than the body condition of cows in gaushalas, where over half of the cows had marginally lower than normal BCS [6]. Again, this may be due to the possibility of sick cows being culled in Kerala. However, the prevalence of good body condition in Kerala may be lower in comparison to Western countries, as the percentage of cows recorded as thin (12.2%) is greater than the percentage of very lean cows recorded on UK farms (5%) [32].

The cow hygiene scores suggest that the lying environment was not being adequately cleaned: over half the cows were dirty, and of the dirty cows, 41.2% had the highest dirtiness score (>40 cm of manure). Poor hygiene is a problem in tie-stalls, as the cow may defecate in the area where she lies due to her restricted movement; this exposure to manure and other dirt can lead to increased risk of mastitis and lameness [20]. In addition, the welfare of cows with soiled resting areas is further reduced because, besides the risk to their health, they are denied a resource (a clean, dry resting area) that they “want”: cows show a clear preference for a dry (clean) lying surface compared to one that is wet due to the elements, faeces and/or urine [33]. Good welfare, according to Dawkins [34], is defined as animals that are “healthy and have what they want”. In comparison, cows in traditional shelters in India were found to have higher cleanliness levels, which the authors concluded were a result of high labour input into cleaning [6]. 

In total, 68.9% of cows had either hair loss, a lesion or swelling; of these, 67.1% had hair loss. This is a similar finding to the prevalence of hair loss in cows in gaushalas, where over half had hair loss (53.2%) and body lesions (56.0%) [6]. The hair loss and body lesions of cows in the gaushalas were mainly caused by interaction with sharp metal on gates, broken mangers, broken edges of walls and barbed wire fencing. In addition, the area per cow in the gaushalas was small, which increased the risk of them sustaining injuries against their surroundings. Restriction of movement combined with hazards in the environment could explain why most cows in this study had hair loss. The prevalence of hair loss in this study is lower than the prevalence of hock hair loss (81.5%) to some degree (43.7% mild, 25.1% moderate, 12.6% severe) found on a large sample of dairy cows in the Midlands region of the UK, a high prevalence which is in agreement with previous UK figures [35]. In that study, hair loss was more prevalent in cubicle housing compared to straw yards, again suggesting that interaction with the physical environment, i.e., cubicle stalls leads to more skin friction and so hair loss [36]. 

Measurements from the Welfare Quality® and other protocols were carried out in the current study without any practical difficulties, suggesting that they are viable for use on Indian dairy farms ranging in size from traditional smallholdings to larger commercial operations. The only exception to this is the behavioural assessment of cows that are close-tied or tethered, due to the limitation of their movement and ability to display normal or even abnormal behaviour [20].

## 5. Conclusions

This study has revealed serious issues of dairy cow welfare associated with husbandry practices that are likely to impact on millions of animals across India. The main welfare issues identified were the behavioural restrictions associated with the use of tie-stall systems (close-tied cows), limited access to water and quality feed and fodder, heat stress, poor hygiene and hair loss. It is likely that in addition to the welfare impact, productivity was impaired by restriction of food and water. Further studies which expand on the sample size and geographic scale of this study are needed to support these findings. Future work should focus on encouraging dairy farmers in India to improve the welfare of their dairy cattle by: ceasing to tie and tether cattle, or at least providing tied and tethered cattle with exercise opportunities; providing unlimited access to drinking water and a readier supply of food (especially quality green forage/fodder); cleaning housing more frequently; providing strategies to prevent heat stress; breeding cattle suited to environmental conditions and with increased resistance to heat stress; carrying out welfare assessments more regularly using a validated protocol, and rectifying the causes of poor welfare. These changes could improve the welfare of tens of millions of cattle.
